# Supplementary figures and images for: Integrin–ECM interactions and membrane-associated Catalase cooperate to promote resilience of the Drosophila intestinal epithelium
Source: PLoS Biol. 2022 May 6;20(5):e3001635. doi: 10.1371/journal.pbio.3001635 (PMC9116668; doi:10.1371/journal.pbio.3001635)

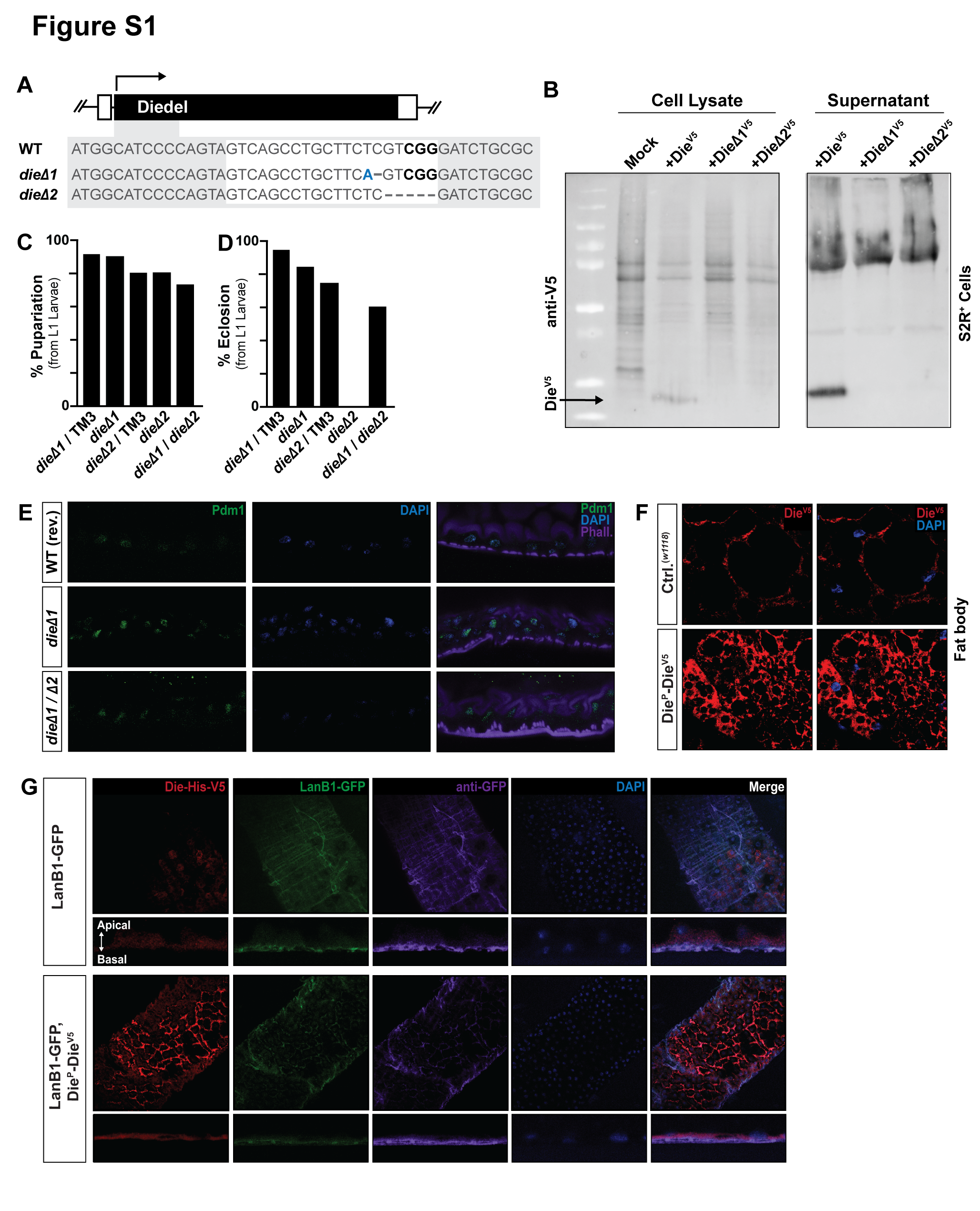

Supplement: S1 Fig — (A) Genomic DNA sequence alignment of wild-type (WT) Diedel and CRISPR-Cas9–induced Diedel mutants (dieΔ1 and dieΔ2). White box highlights gRNA sequence; nucleotides represented in blue highlight a mismatch; dashed lines highlight nucleotide deletions. (B) Western blot with anti-V5 antibody of cell lysate or conditioned media (supernatant) from S2R+ cells either nontransfected (Mock) or transfected with a plasmid containing Die-V5, or DieΔ1-V5, or DieΔ2-V5. Die-V5 band is highlighted by black arrow. (C) Histogram representing percentage of L1 larva (from different Diedel mutant genotypes) reaching pupariation. (D) Histogram representing percentage of L1 larva (from different Diedel mutant genotypes) reaching adult stage (eclosion). (E) Pdm1 immunostaining of dissected posterior midgut from control (WT, revertant [rev.]), Diedel mutant homozygote (dieΔ1/dieΔ1), and Diedel mutant trans-heterozygote (dieΔ1/dieΔ2) flies; stained with anti-Pdm1 (green), Phalloidin (Phall.; purple), and DAPI (blue). (F) Die-V5 levels in dissected Drosophila fat bodies from controls (negative control; w1118) and w1118; DieP-Die-V5 / DieP-Die-V5 transgenic flies; stained with anti-V5 antibody (Die-V5; red) and DAPI (blue). (G) Die-V5 and Laminin B1 (LanB1) immunostaining of dissected posterior midguts from endogenously tagged Laminin B1 (LanB1-GFP) transgenic flies and LanB1-GFP/DieP-Die-V5 flies; bottom panel represents cross-section (apical–basal polarity is highlighted with arrow); stained with anti-His (Red), anti-GFP (purple), and DAPI (blue). The data underlying the graphs shown in S1C and S1D Fig can be found in S1 Data. (TIF) [file pbio.3001635.s001.tif]

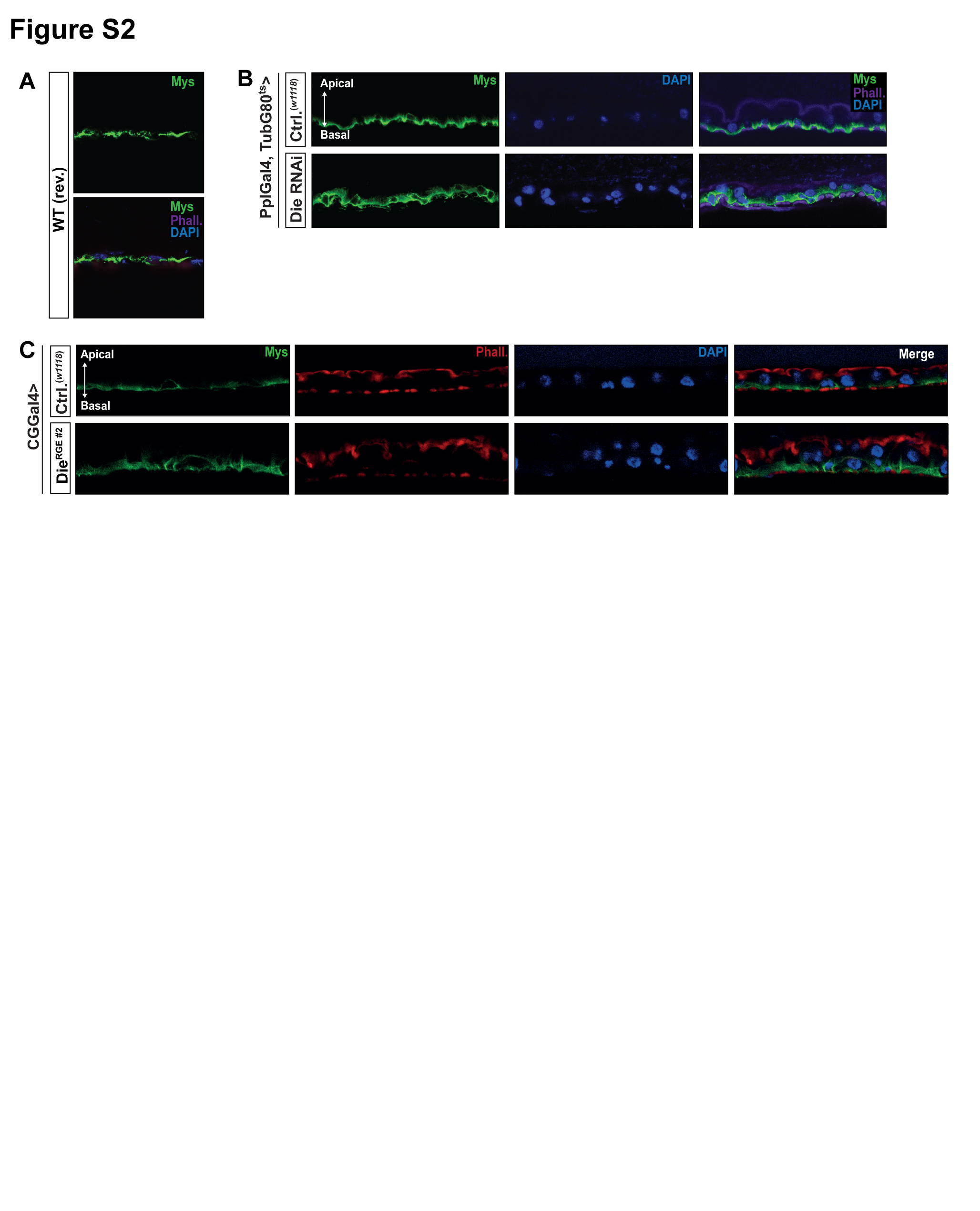

Supplement: S2 Fig — (A) Integrin Mys immunostaining of dissected posterior midgut from control (WT, revertant [rev.]) flies; panels represent cross-sections (apical–basal polarity is highlighted with arrow); stained with anti-Mys (green), Phalloidin (Phall.; purple), and/or DAPI (blue). (B) Integrin Mys immunostaining of dissected posterior midgut from control flies (w1118; PplGal4,TubG80ts) and flies with conditional (adult-specific) fat body attenuation of Diedel (w1118; PplGal4,TubG80ts / UAS-Die RNAi) after 7 days at 29°C; cross-sections (apical–basal polarity is highlighted with arrow); stained with anti-Mys (green), Phalloidin (Phall.; purple), and/or DAPI (blue). (C) Integrin Mys immunostaining of dissected posterior midguts from control flies (w1118; CGGal4) and flies with fat body expression of Diedel RGE (w1118; CGGal4 / UAS-DieRGE#2: transgenic line 2), stained with anti-Mys (green), Phalloidin (Phall.; red), and/or DAPI (blue). (TIF) [file pbio.3001635.s002.tif]

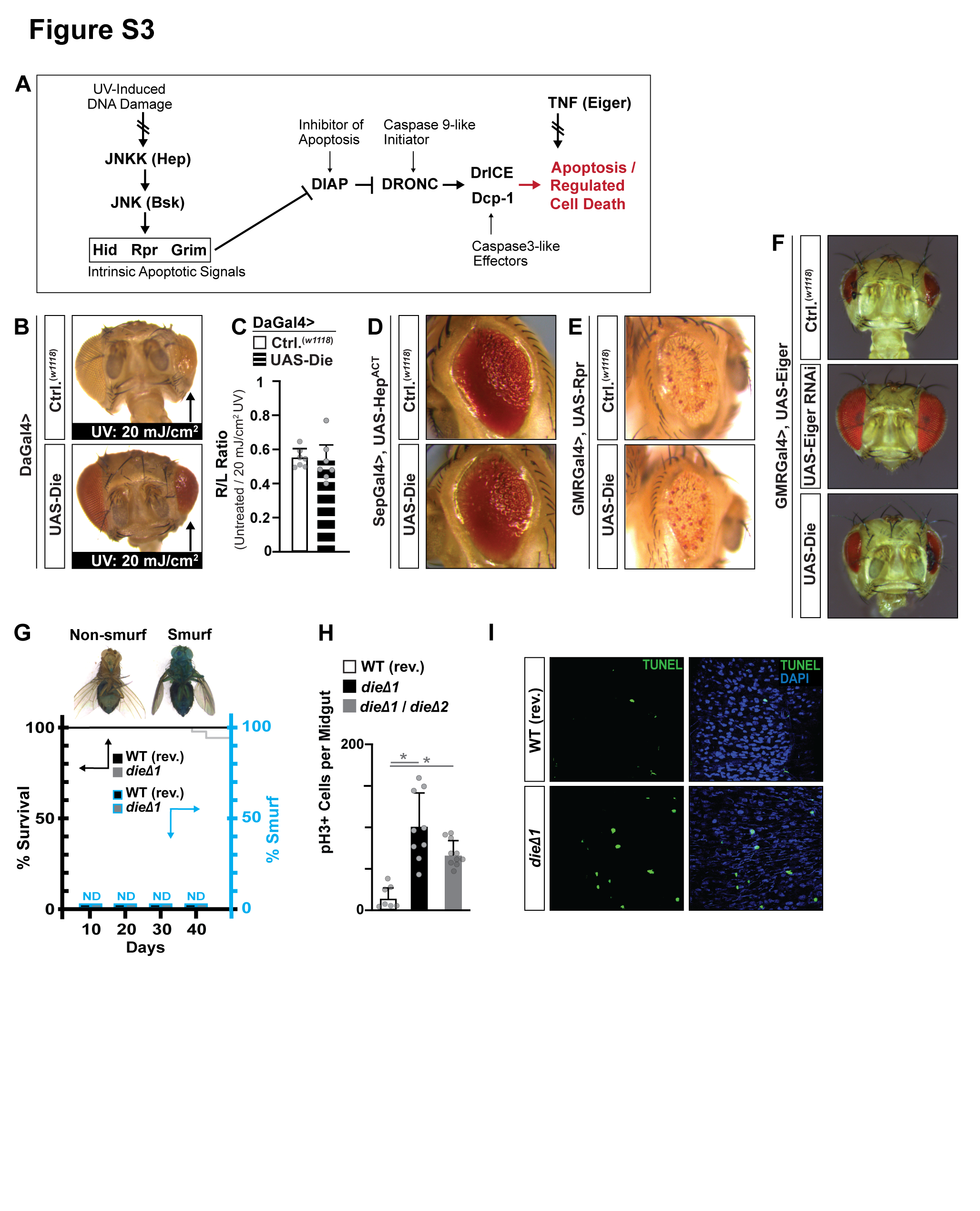

Supplement: S3 Fig — (A) Schematic of the intrinsic and extrinsic (TNF) apoptosis-induced RCD pathway in Drosophila. (B) UV induced retinal apoptosis. Photomicrographs of adult eyes from control flies (w1118) or flies ubiquitously overexpressing Diedel (w1118; DaGal4 / UAS-Die) During pupal development, retina were either UV irradiated (20 mJ/cm2, right eye; black arrow) or not (left eye). (C) Histogram representing the ratio between the eye area of the irradiated eye (right, R) to the non-irradiated (untreated) eye (left, L) of the genotypes described above. Bars represent mean ± SE, n = 7. (D) Constitutive activation of JNKK (UAS-HepACT) in the developing fly retina (SepGal4; expressed in photoreceptors and cone cells) results in a “rough” eye phenotype induced by apoptosis. Overexpressing Diedel (w1118; SepGal4, UAS-HepACT / UAS-Die) does not change this phenotype compared to controls (w1118; SepGal4, UAS-HepACT). (E) Overexpression of Reaper (UAS-Rpr) in the developing fly retina (GMRGal4) results in an apoptosis-induced cell death. Overexpressing Diedel (w1118; GMRGal4, UAS-Rpr / UAS-Die) does not change this phenotype compared to controls (w1118; SepGal4, UAS-Rpr). (F) Overexpression of Eiger (UAS-Eiger) in the developing fly retina (GMRGal4) results in an apoptosis-induced cell death rescued by Eiger inhibition (GMRGal4, UAS-Eiger / UAS-Eiger RNAi). Overexpressing Diedel (w1118; GMRGal4, UAS-Eiger / UAS-Die) does not change this phenotype compared to controls (w1118; GMRGal4: UAS-Eiger). (G) Intestinal barrier dysfunction (“Smurf” assay) in control (WT, revertant [rev.]), Diedel mutant homozygote (dieΔ1/dieΔ1) during aging. ND, nondetected, n = 100 flies/group. (H) Quantification of ISC proliferation (mitoses per whole dissected midgut); assayed by anti-pH3 immunostaining in control (WT, revertant [rev.]), Diedel mutant homozygote (dieΔ1/dieΔ1) and Diedel mutant trans-heterozygote (dieΔ1/dieΔ2) flies. Bars represent mean ± SE, n = 10–15. *P ≤ 0.05 (Student t test). (I) Representa [file pbio.3001635.s003.tif]

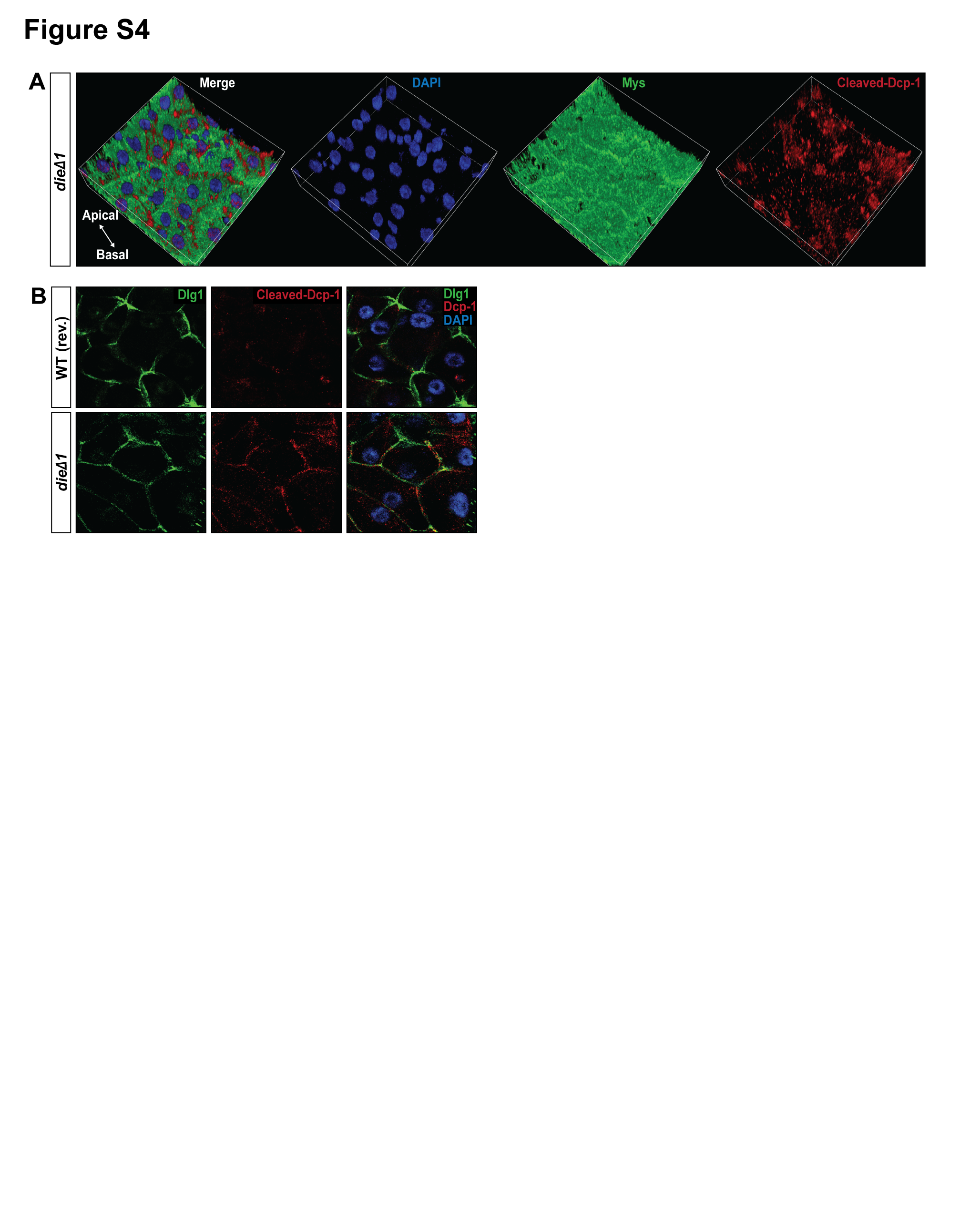

Supplement: S4 Fig — (A) Integrin Mys and Caspase (cleaved Dcp-1) immunostaining of dissected posterior midgut of control (WT, revertant [rev.]) and Diedel mutant (dieΔ1/dieΔ1) flies; stained with anti-Mys (green), anti-Dcp-1 (Cleaved Dcp-1; red), and DAPI (blue). Images represent 3D Confocal Z-Stack representation of Integrin Mys and Cleaved Dcp-1 localization. Apical–basal polarity/arrangement in sections is highlighted. (B) Disc large 1 (Dlg1; septate junction marker) and Caspase (cleaved Dcp-1) immunostaining of dissected posterior midgut of control (WT, revertant [rev.]) and Diedel mutant (dieΔ1/dieΔ1) flies; stained with anti-Dlg (green), anti-Dcp-1 (Cleaved Dcp-1; red), and DAPI (blue). (TIF) [file pbio.3001635.s004.tif]

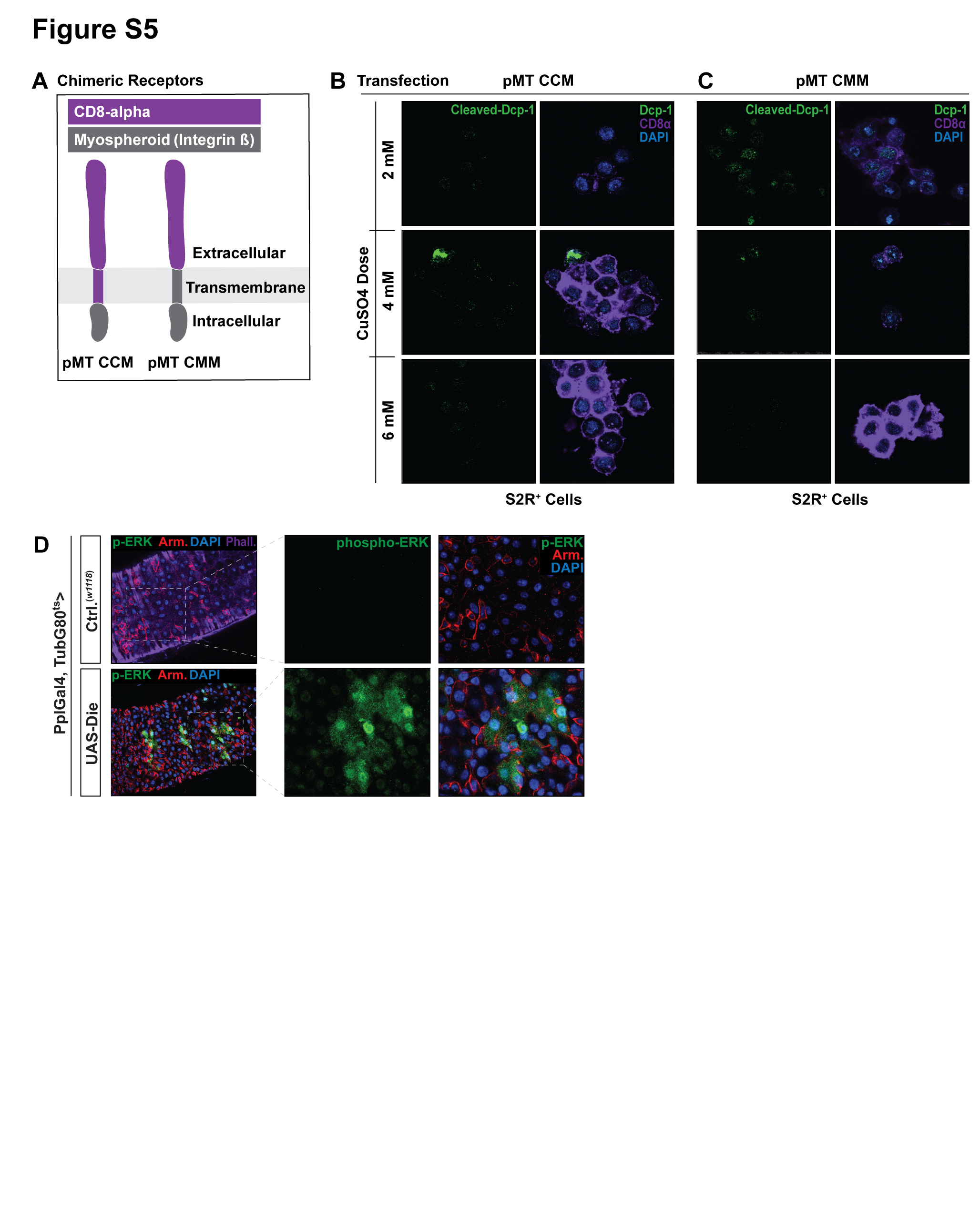

Supplement: S5 Fig — (A) Schematic of CD8-alpha and Integrin Mys chimeric receptors. pMT CCM expresses the extracellular and transmembrane region of the mouse CD8-alpha and the intracellular region of Integrin Mys; pMT CMM expresses the transmembrane and intracellular region of Integrin Mys and the extracellular region of CD8-alpha (B-C) Cleaved DCP1 and CD8-alpha immunostaining of S2R+ cells transfected with chimeric receptors (B) pMT CCM and (C) pMT CMM. Chimeric receptor (plasmid) expression was induced by CuS04 at 3 concentrations, 2 mM, 4 mM, and 6 mM, and stained with anti-cleaved DCP1 (green), anti-CD8-alpha (Cleaved Dcp-1; red), and DAPI (blue). (D) phospho-ERK immunostaining of dissected posterior midgut from control flies (w1118; PplGal4,TubG80ts), flies with conditional (adult-specific) fat body attenuation of Diedel (w1118; PplGal4,TubG80ts / UAS-Die RNAi), or conditional (adult-specific) fat body overexpression of Diedel (w1118; PplGal4,TubG80ts / UAS-Die) after 5 days at 29°C; stained with anti-phoso-ERK (green), anti-Arm (armadillo; red), and DAPI (blue). (TIF) [file pbio.3001635.s005.tif]

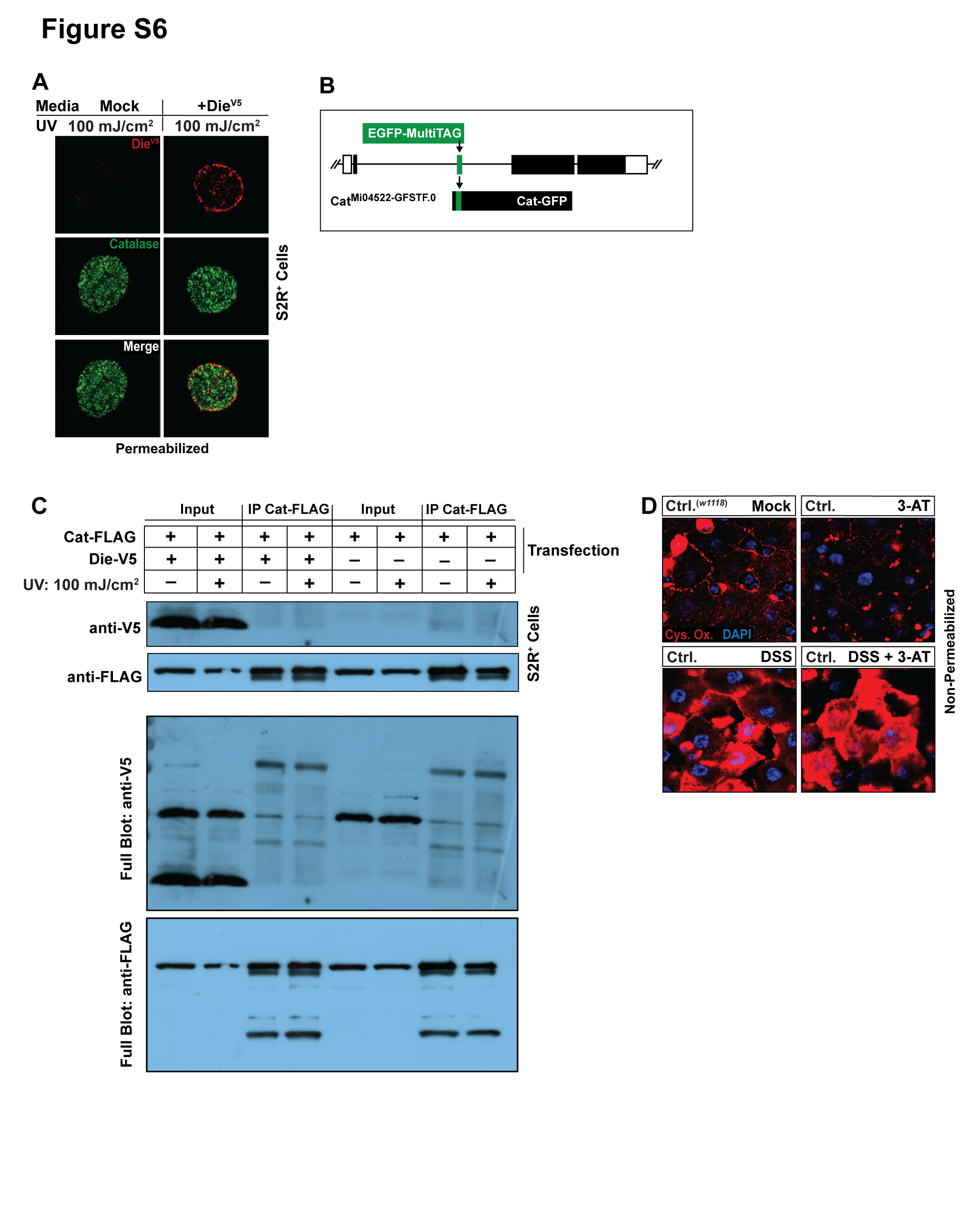

Supplement: S6 Fig — (A) Catalase and anti-V5 immunostaining of S2R+ cells after UV exposure (100 mJ/cm2); cells treated with Die-V5 conditioned media (supernatant) or Mock (control); stained with anti-V5 antibody (Die-V5; red) and anti-Catalase (green). Cells were with detergent in order to visualize intracellular proteins. (B) Schematic representation of the EFGP-multiTAG insertion into the Drosophila Catalase gene loci. (C) Co-immunoprecipitation of Die-V5 and Cat-FLAG in S2R+ cells transfected with pAC.5.1 Cat-Flag or cotransfected with pAC.5.1 Cat-Flag and pAC5.1 Die-V5; +/− UV: 100 mJ/cm2. Western blot using anti-Flag antibody and anti-V5 antibody. Bottom panels display entire images of western blots. (D) Membrane/ECM cysteine oxidation of dissected posterior midguts of w1118 (Ctrl.) flies; DSS treatment/feeding (4% DSS), or mock treatment, or cotreatment/feeding with a Catalase inhibitor (DSS + 3-AT); stained with anti-Cysteine (oxidized, Cys. Ox; red) and DAPI (blue). Immunostaining was performed in nonpermeabilized conditions to visualize membrane/ECM cysteine oxidation. Flies were fed (or Mock treated) DSS or DSS + 3-AT for 2 days. (TIF) [file pbio.3001635.s006.tif]

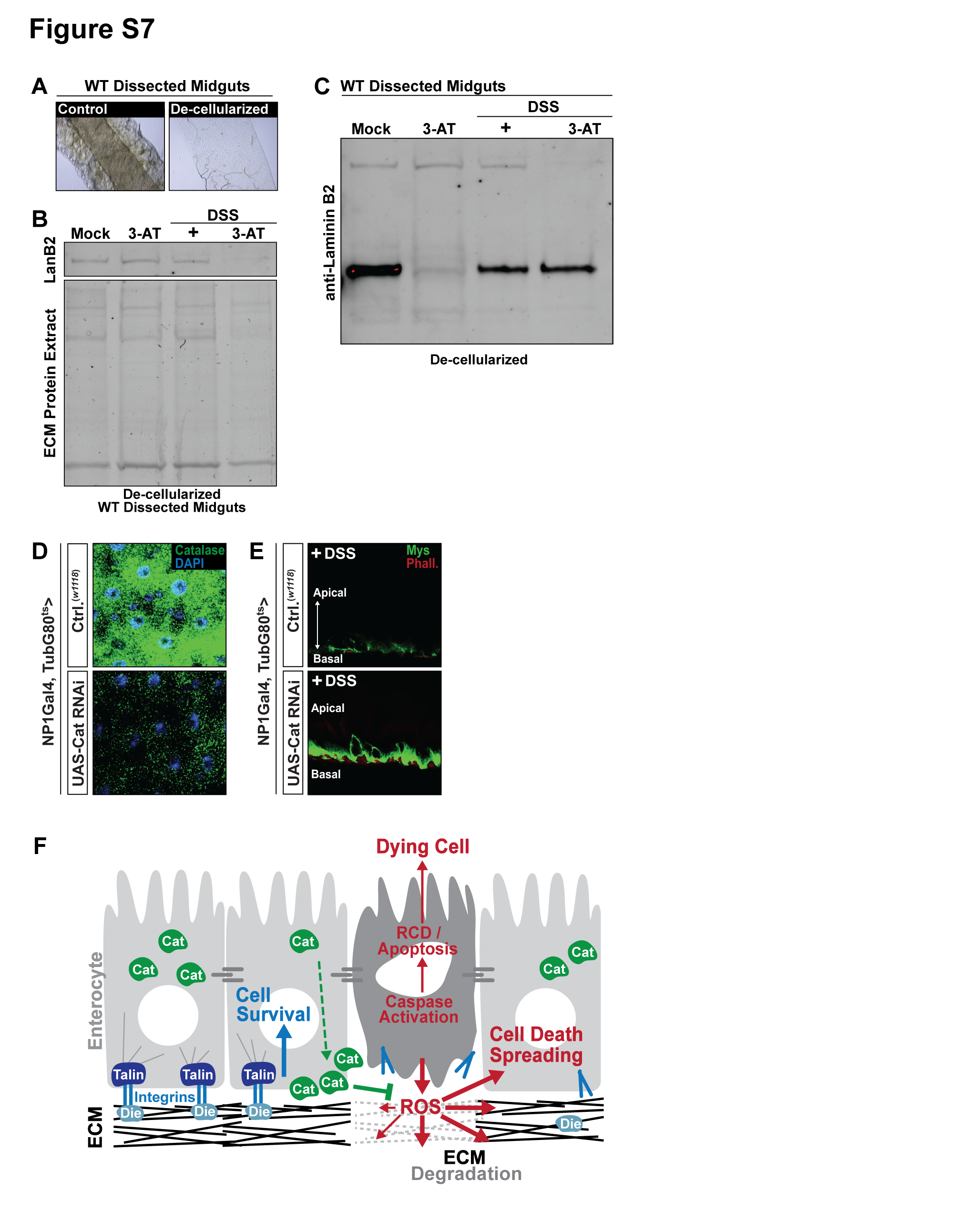

Supplement: S7 Fig — (A) Brightfield microscopy images of w1118 (WT; control) Drosophila posterior midguts before and after decellularization. (B) Western blot with anti-Laminin B2 antibody (LanB2, top panel) and ECM protein composition shown by stain free gel (bottom panel); after midgut decellularization. w1118 (WT; control) midguts were dissected after DSS treatment/feeding (4% DSS, +), or mock treatment, or cotreatment/feeding with a Catalase inhibitor (DSS + 3-AT). Flies were fed (or Mock treated) DSS or DSS + 3-AT for 2 days. (C) Entire image of western blot; related to S7B Fig top panel. Western blot with anti-Laminin B2 antibody after midgut decellularization.(D) Catalase immunostaining of dissected posterior midgut of control flies (w1118; NP1Gal4, tubG80ts) or flies with adult enterocyte-specific inhibition of Catalase (w1118; NP1Gal4, tubG80ts / UAS-Catalase [Cat] RNAi); stained with anti-Catalase (green) and DAPI (blue). Immunostaining confirms RNAi efficacy. (E) Integrin Mys immunostaining of dissected posterior midgut of control flies (w1118; NP1Gal4, tubG80ts) or flies with adult enterocyte-specific inhibition of Catalase (w1118; NP1Gal4, tubG80ts / UAS-Catalase [Cat] RNAi); midguts were dissected after DSS treatment/feeding (+ 4% DSS) and stained with anti-Mys (green) and Phalloidin (Phall.; red). Panels represent cross-sections (apical–basal polarity is highlighted with arrow). (F) Proposed model depicting the role of Diedel–Integrin–ECM interactions and membrane-associated Catalase in the regulation of extracellular ROS to promote epithelium resilience. (TIF) [file pbio.3001635.s007.tif]
