# Supplementary material for: Integrin–ECM interactions and membrane-associated Catalase cooperate to promote resilience of the Drosophila intestinal epithelium
Source: PLoS Biol. 2022 May 6;20(5):e3001635. doi: 10.1371/journal.pbio.3001635 (PMC9116668; doi:10.1371/journal.pbio.3001635)

Related to Fig. 2 c

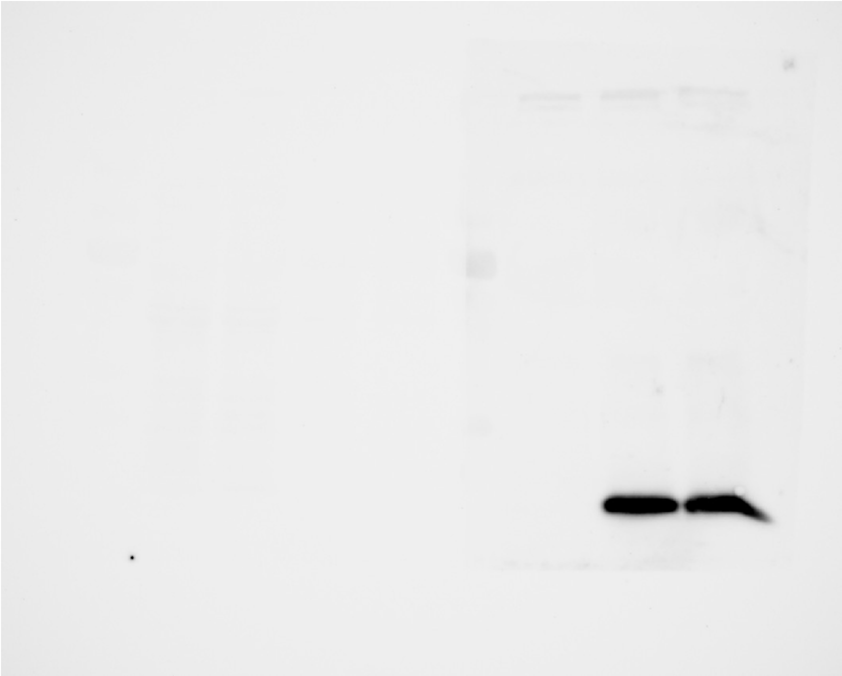

Over-exposition

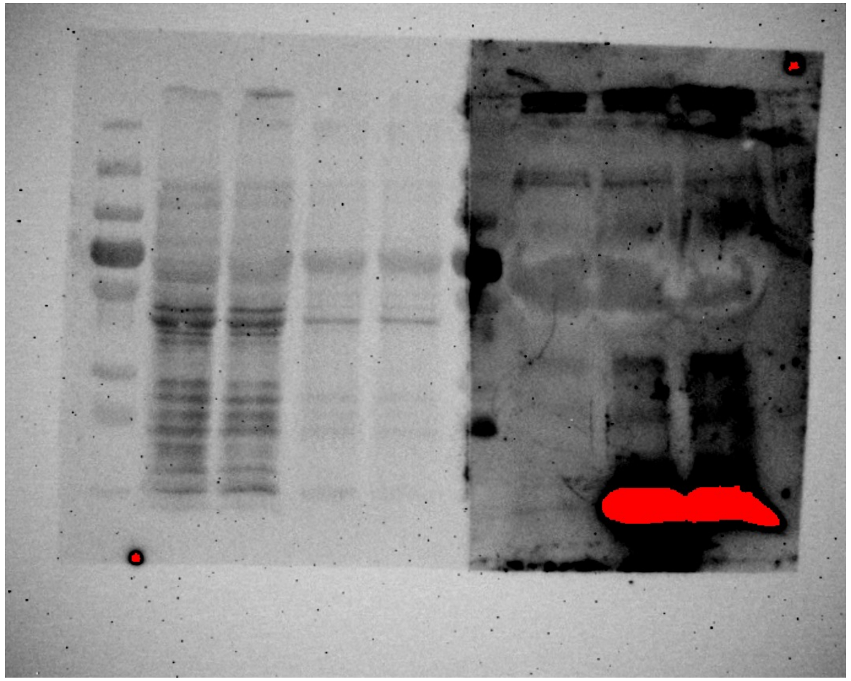

Related to Fig. 4 A

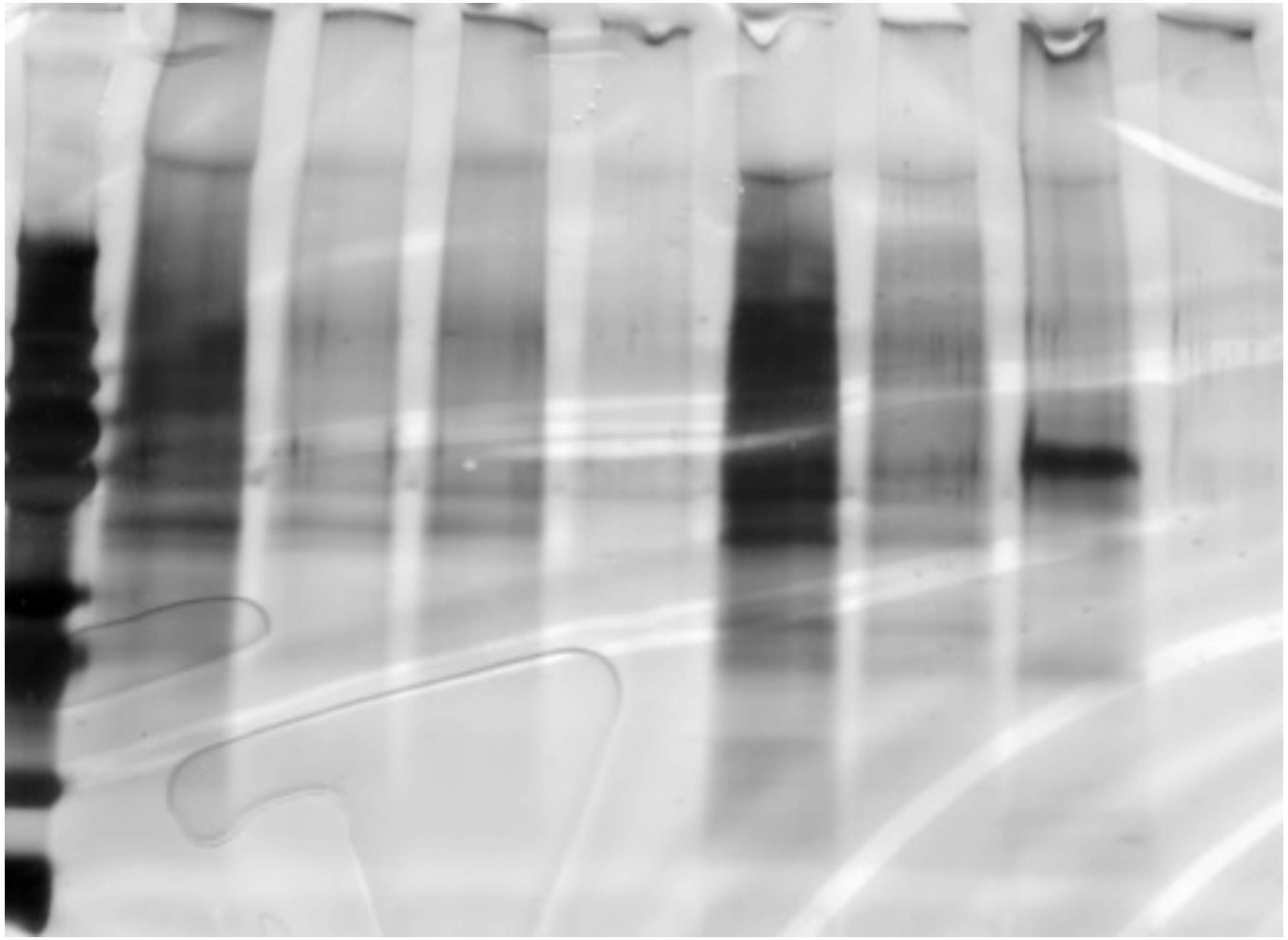

Related to Fig. S1 B

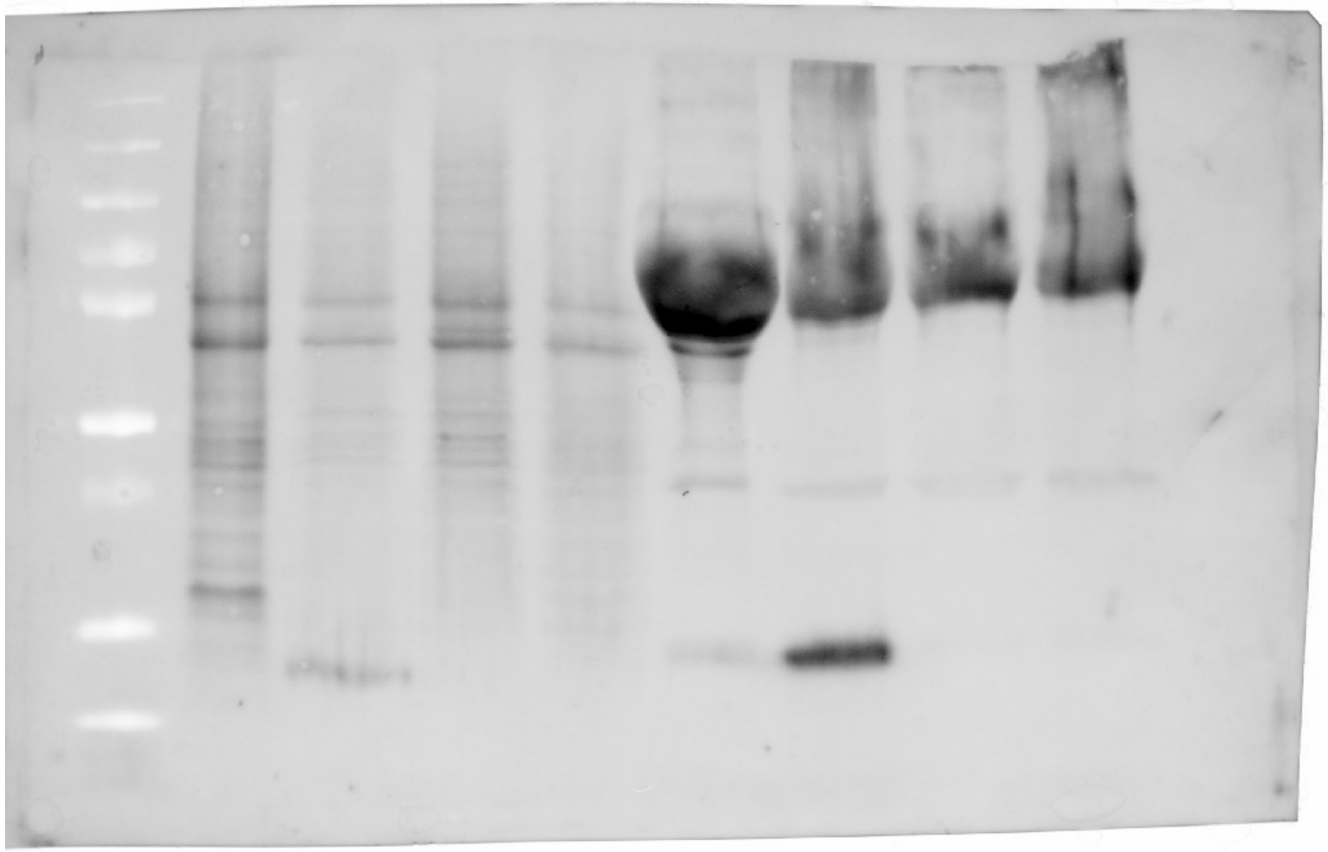

Related to Fig. S6 C

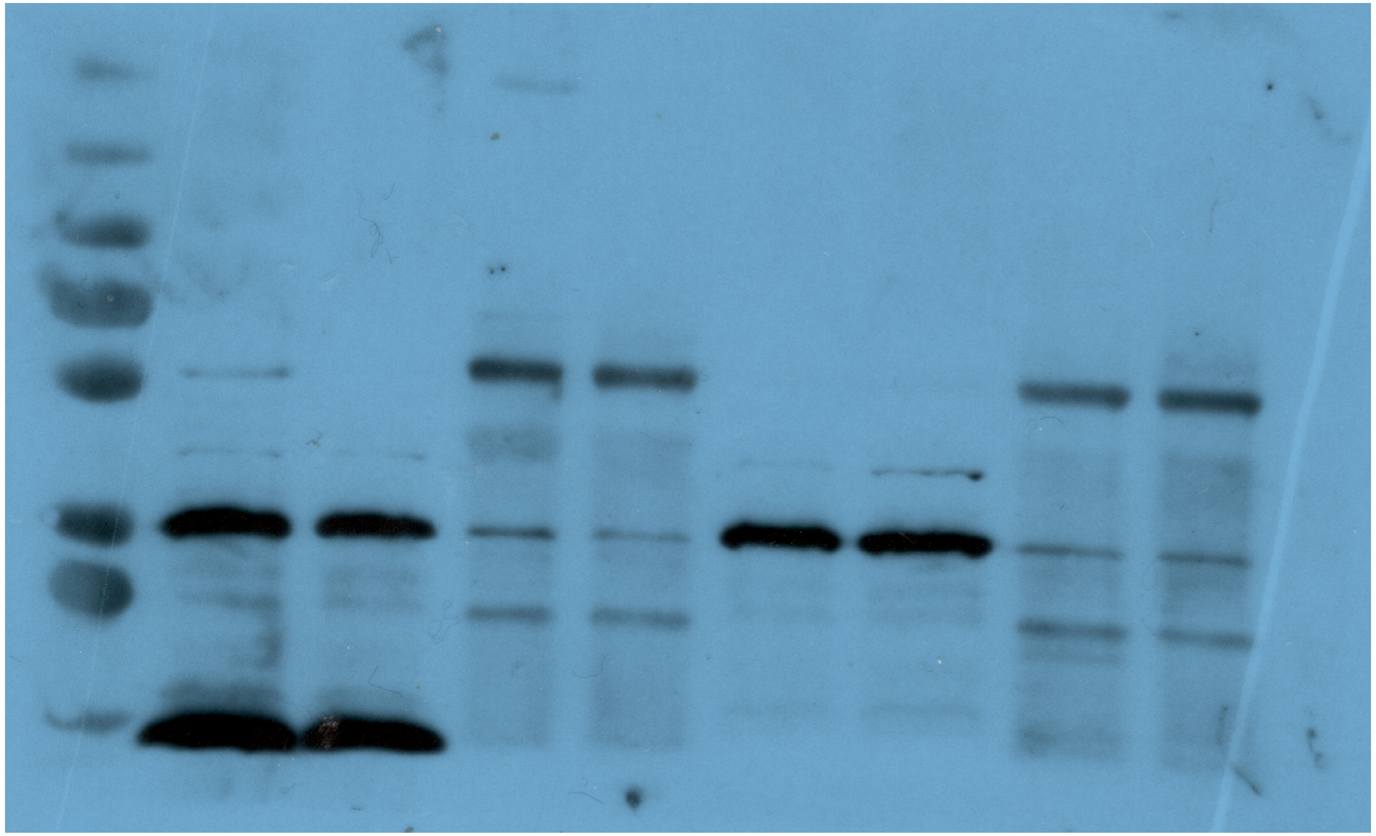

Related to Fig. S6 C

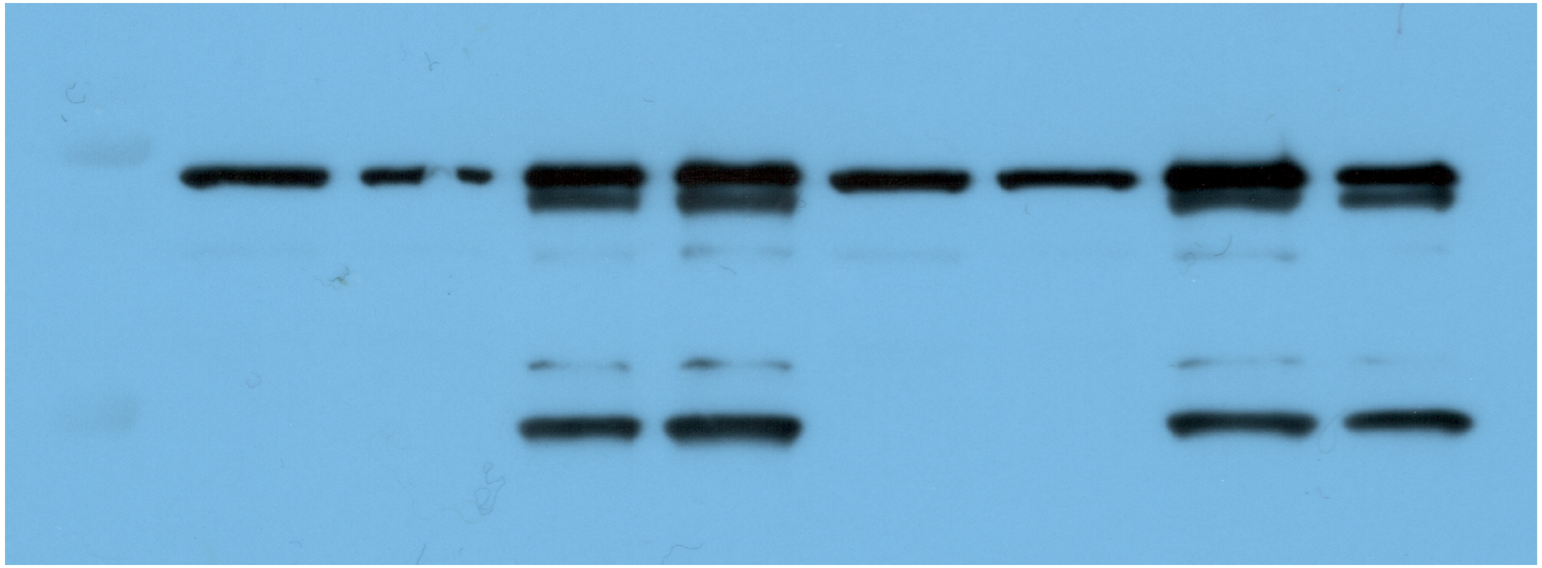

Related to Fig. S7 B-C

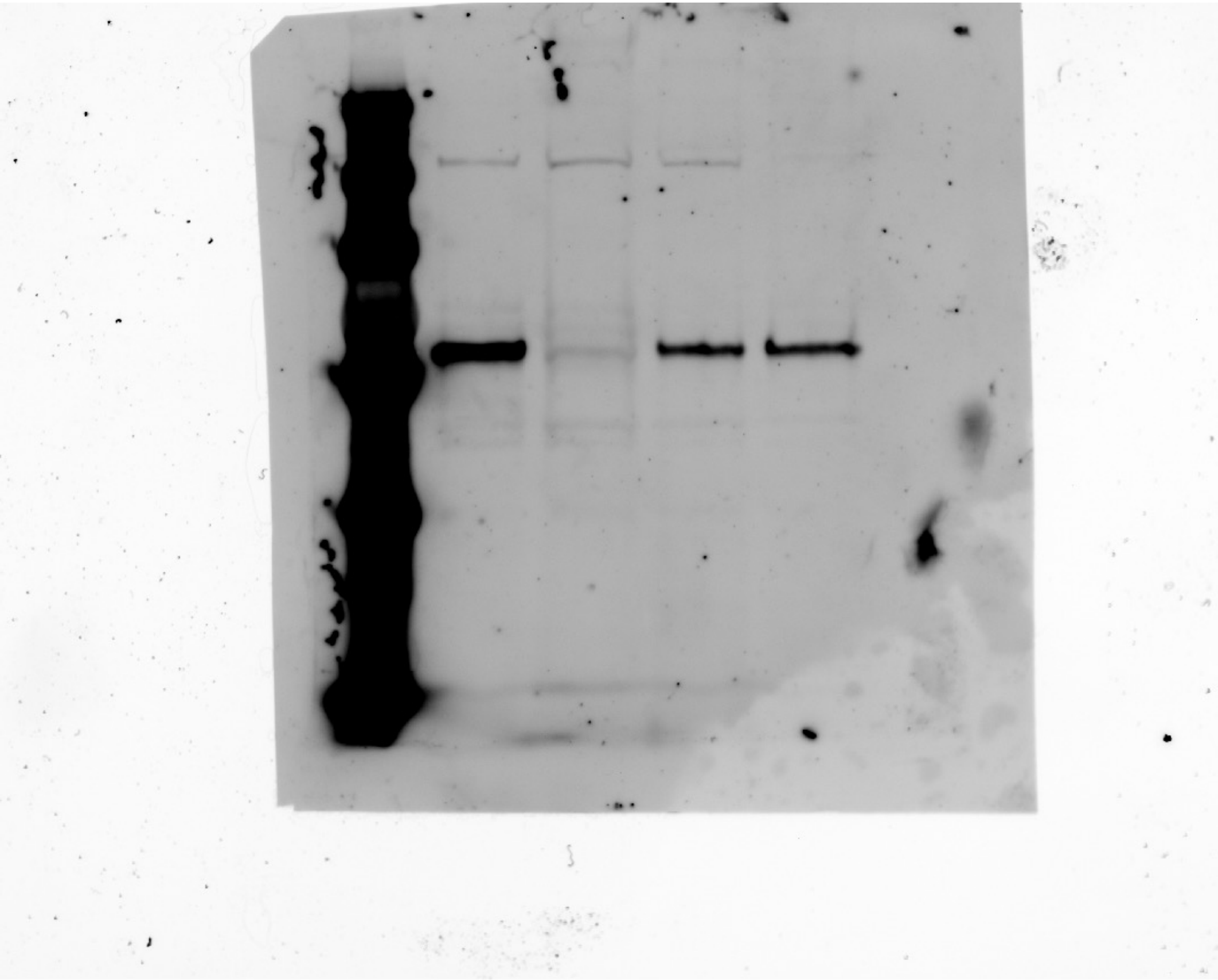

Related to Fig. S7 B

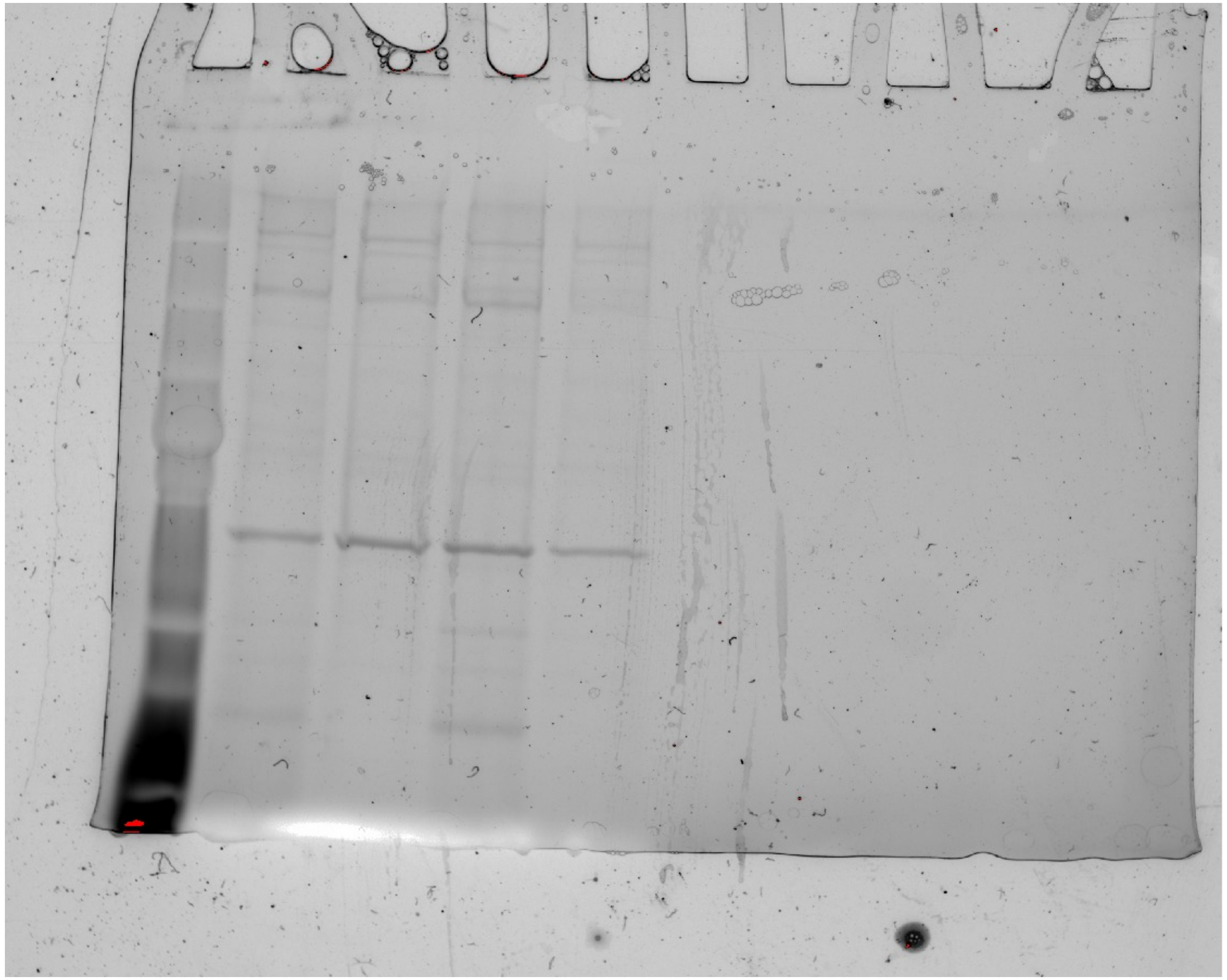

Supplement: S1 Raw Images — (PDF) [file pbio.3001635.s011.pdf]
