# Supplementary material for: Integrin–ECM interactions and membrane-associated Catalase cooperate to promote resilience of the Drosophila intestinal epithelium
Source: PLoS Biol. 2022 May 6;20(5):e3001635. doi: 10.1371/journal.pbio.3001635 (PMC9116668; doi:10.1371/journal.pbio.3001635)
Supplement: Attachment — Submitted filename: Response to Reviewers.docx [file pbio.3001635.s012.docx]

We would like to thank the reviewers for both positive feedback and constructive criticism. In this revised version of the manuscript and the following response to reviewer critiques, we are including additional data and control experiments, as well broadened explanations / discussions, in order to address the concerns that were highlighted. We believe that these new observations have strengthened and expanded the phenotypes, mechanisms, and hypotheses presented in the manuscript.

Reviewers’ comments

Rev.1:
This study identifies a novel mechanism of the Drosophila gut tissue against apoptotic cell death. It revealed how Diedel secreted from the fat body protects enterocytes via its binding ability to Integrin through the RGD domain. Through the interactome analysis, the authors further identified that Catalase is bound at the membrane and preserve the gut tissue. This relocalisation of Catalase inhibits the spreading of the apoptosis by reducing ROS as an adaptive stress response. The study has been done well and the manuscript are carefully written honestly. Overall, the data provided support the conclusion, and their finding is interesting.

Major comments,

1, Throughout the manuscript, the authors use either whole body mutant (It is nice that the authors characterise the phenotype well) or RNAi, by which they cannot deny the possible effect on the development of the gut. They need to do some adult-specific manipulation, which would tell more how adult homeostasis is perturbed by the temporal loss of Diedel/Catalase function in the adult gut. The time course analysis would further strengthen the description of the phenotype.

We have **added new data** utilizing adult-and-tissue-specific attenuation of Diedel (PplGal4,tubGal80TS>UAS-Die RNAi) to complete the other genetic models. Adult (temporal)-specific attenuation of Diedel also leads to Integrin mis-localization and enterocyte detachment from the midgut basement membrane **(Fig. S2B).**

Furthermore, in our previously published manuscript, we used multiple adult-specific genetic drivers (mainly GeneSwitch) to explore the role of Diedel in regulating cell death in the midgut (**Mlih et. al., PLoS Biology, 2018**).

2, It is not clear for this reviewer how Diedel, Integrin, or catalase localisation is regulated.

We agree that the initial submission did not clearly integrate Diedel-Integrin-ECM interactions with membrane-associated Catalase. To this end, we have added **new text, discussion, and data (as well as updated the title)** to better highlight the synergy between these functions.

First, we show that adult-specific Diedel over-expression (PplGal4,TubGal80^ts^>UAS-Die) leads to enhanced phospho-ERK immunostaining in midgut enterocytes (**Fig. S5A-D**), suggesting that Diedel-Integrin-ECM interactions likely shape the balance of cell death by, in part, promoting cell survival. Next, we show that while Catalase is present at membranes enriched for Diedel-Integrin-ECM interactions, co-immunoprecipitations in native (non-crosslinking) conditions highlight that Diedel and Catalase do not directly interact (**Fig. S6C**), and thus Catalase is likely part of a larger membrane interactome, associated with Integrin-ECM interactions, that shapes cell death and survival responses. We also show that Catalase re-localizes to membranes in cells adjacent (‘neighbor’) to dying cells in the midgut in vivo using MARCM ‘undead’ clone analysis (**Fig. 5B**). Finally, utilizing a neutralizing antibody, we show that Catalase, and thus likely extracellular and/or membrane-associated Catalase, is necessary to prevent cell death spreading and promote cell survival driven by Diedel *in vitro* (**Fig. 6C-D**). Coupled with our other findings, these data highlight that Diedel-Integrin-ECM interactions likely promote survival of ‘neighboring’ cells, allowing these cells to endure stress an promote Catalase re-localization at membranes to eliminate extra-cellular ROS (**Fig. 5-6**). Thus, these mechanisms likely work in synergy to regulate cell death spreading and ECM degradation in epithelial tissues during stress.

2-1, Fig.1F suggests the binding of Die to gut (in normal condition), but for S2 cells, the Die binding is UV-dependent (Fig. 3A, 4D). How has UV-stimulation enabled Die to be at the membrane?

As the primary barrier epithelium, the Drosophila midgut endures a basal/normal level of stress that drives minimal turnover (enterocyte death - stem cell proliferation/differentiation and enterocyte – enterocyte replacement, (**Liang et al. Nature 2017**)). This is likely why Diedel is prevalent at the basement membrane of the midgut even in the absence of acute stress.

However, S2R+ cells are immortalized. In order to study cell death, ‘stress’ must be added to the system. We considered increasing the passage number of the cells, causing them to go through senescence, however this impacts reproducibility of all experiments. UV treatment was chosen for 2 reasons: (I) it provides consistent results in cell death induction, and (II) it has previously been used to explore ROS-based signaling mechanisms *in vitro* in S2 cells (which we also know is important for Diedel-dependent control of cell-death spreading (**Fig. 5-6**)). Thus, UV acts as a ‘stressor’ in S2 cells that influences cell membranes and potentiates Deidel binding. It is very likely that a variety of other types of ‘stressors’ can also potentiate Diedel binding to these cells *in vitro*.

2-2, Why Integrin is not identified in the interactor of Die-V5? Related to 2-1, Is it because S2 cells do not express enough integrins? Please discuss this point.

Indeed, S2R+ cells don’t express (both in quantity and variety) many types of Integrins. While the Integrin Mys is expressed in S2R+ cells, its heterodimer and the Integrin we find necessary to bind Deidel (If) is less abundant.

Also, in general, pulling-down Integrin receptor heterodimers is biochemically very challenging, and requires unique assays and techniques.

2-3, In Fig. 5, the authors claimed that Catalase is localised to the membrane upon DSS feeding, but it is interesting whether Die is required for this phenotype. Whether Die mutant fails to show this Catalase relocalisation is vital step to draw the conclusion.

At this point, we do not believe the Diedel is a molecular driver or anchor that is molecularly/biochemically essential for Catalase re-localization (**see Response to Point 2 as well**), and that these mechanisms likely work in synergy.

To this end, we have **included additional data** that we believe helps better understand why Catalase is enriched at membranes when Diedel is present. First, we show that adult-specific Diedel over-expression (PplGal4,TubGal80^ts^>UAS-Die) leads to enhanced phospho-ERK immunostaining in midgut enterocytes (**Fig. S5A-D**), suggesting that Diedel-Integrin-ECM interactions likely shape the balance of cell death by, in part, promoting cell survival. We also show that Catalase re-localizes to membranes in cells adjacent (‘neighbor’) to dying cells in the midgut in vivo using MARCM ‘undead’ clone analysis (**Fig. 5B**). Lastly, Diedel alone can’t drive Catalase to membranes *in vitro* or *in vivo* (see **Fig. 5C** as an example). Thus, during stress, Diedel-Integrin-ECM interactions likely shift the balance towards cell survival, which helps us visualize Catalase at membranes. Over-expressing or treating cells with Diedel enriches this even further by increasing the number of cells that survive and attenuating cell death spreading. Integrin-ECM interactions and membrane-associated Catalase are likely to work in synergy to influence epithelial resilience.

*That exact/precise molecular mechanism by which Catalase re-localizes to cellular membranes remains unclear, and will be explored in future studies.*

3, One of the most attractive points of the manuscript would lie in the "cell death spreading" protected by Die. In Fig. 3B-D, together with Fig. S2, the authors tried to show the non-cell-autonomous killing of the cell is affected by Die. However, it is not directly tested whether this is the case in the gut tissue. In S2 cells, the type of cell death induced here is not well understood (is it apoptosis? through what kind of secreted protein?). It is technically not difficult to test the contribution of cell death-inducing cell death by overexpressing rpr/hid in the gut in a clonal manner. Also, they can probably use the model of apoptosis-induced apoptosis in the wing discs (for example, Perez-Garijo et al., elife, 2013).

First, this elegant clonal system describing ‘undead’ cells expressing Hid/Rpr (initially performed in wing discs, Perez-Garijo et al., eLife, 2013) does represent an intriguing tool to study cell death spreading in the adult midgut. However, we would have been unable to reconstruct the entire MARCM system in a Diedel CRISPR mutant background (that involves precise PCR sequencing/genotyping and screening at every genetic construction step) within a normal revision timeline. This is because we are not simply making Diedel mutant clones, but rebuilding the entire MARCM system into a genetic background that can only be assessed through sequencing/genotyping and not phenotyping. We were, however, able to use this MARCM system to analyze Catalase membrane localization using Catalase-GFP in heterozygosity (described in other responses, **Fig. 5B**).

Second, it is likely that the regulation of cell death in midgut enterocytes is very different from that in imaginal disc epithelial tissues. Because the function of the gut as barrier is essential for survival of the fly, we show here that even if enterocyte caspase activation is enhanced in the absence of Diedel, the elimination of dying cells is still well controlled **(Fig. 3 and Fig. S4).** We also showed that septate junctions in the epithelium may act as a safeguard to maintain gut barrier function (**Fig. S4B**).

To this end, we turned to the *in vitro* cell death spreading assay (**which we now show** enables apoptosis, TUNEL, **Fig. 6C-D**). We have also **added data to show** that Catalase (and likely membrane-associated Catalase) is required for Diedel-dependent effects on cell death and cell death spreading (**Fig. 6C-D**). Since the only known function of Catalase is to eliminate hydrogen peroxide, it is likely that the secondary signal coming from the dying cell are ROS or ROS-generating factors. Furthermore, it was shown in another study that dying Drosophila cells recruit the oxidase DUOX at cellular membranes to generate extracellular ROS, and that by expressing a human secreted Catalase, cell death inducing proliferation can be attenuated (**Fogarty** **et al. Current Biology 2016**).

We have also attempted to clarify all of this in the text.

4, Did the authors test the expression of Die in the gut?

In Fig. 1F and S1E, they use DieP-DieV5, but they might use fat body Gal4 to express Die V5 to see whether Die is from the fat body. Also, they can use Die-/- plus FB>Die (and the negative control DieRGD) to beautifully show how fat body-derived Die is a key for their phenotype through its binding to Integrin.

In our previously published manuscript (**Mlih et. al., PLoS Biology, 2018**), we generated an *in vivo* reporter for Diedel expression and could only detect induction in the fly thoracic muscle (mild) and in the fat body (strong), which is in accordance with other studies (Lamiable et. al., PNAS, 2016). Furthermore, multiple single cell RNA datasets from the Drosophila midgut also suggest Diedel is not expressed in the gastrointestinal tract (https://www.flyrnai.org/scRNA/gut/). It’s not surprising to see an Integrin ligand/ECM protein mainly expressed in the fat body, as the fat body is the source of other midgut basement membrane proteins (such as Tiggrin and Sparc, (**Fogerty et al. Development 1994; Martinek et al. Dev Genes Evol. 2002**))

Finally, for the revision **we generated transgenic flies** that express UAS-linked Diedel containing the RGD-to-RGE substitution used *in vitro* (UAS-Die^RGE^). Overexpression of Diedel^RGE^ from the fat body (CGGal4>UAS-Die^RGE^) induced disorganization of the midgut epithelium characterized by enterocyte detachment and integrin (Mys) delocaliztion (**Fig. 2F and Fig. S2C**). This phenotype is characteristic of Diedel mutants, suggesting that Diedel^RGE^ coming from the fat body can competitively antagonize normal Diedel (Diedel^RGD^) function.

Rev2: :

This is a review of the manuscript "Membrane-associated Catalase protects Integrin-ECM interactions and promotes resilience of the Drosophila intestinal epithelium" by Mlih and Karpac. In this work, the authors begin by exploring the function of the secreted signal Diedel and end by looking at a potential stress-induced change in catalase localization.

The first part of the paper, on Diedel function, is generally good, and appears well-done. They analyze a Diedel mutant they generated, and then go on to show that Diedel appears to function, at least in part, by binding integrins. I have no significant concerns about this section.

The second part of the paper focuses on catalase localization and oxidative damage to epithelia, with which Diedel is supposed to interact. This part of the paper contains several interesting observations, but these appear to be held together by a series of untested assumptions. A large amount of experimental work would be required to bring the second section up to a publishable standard.

Major issues:

1. One of the major points of the second part of the paper is that the authors observe what appears to be extracellular catalase. The implicit assumption is that catalase is somehow being secreted by stressed epithelial cells. However, it's not clear that this is how this system would have to work: for example, catalase could end up in the extracellular space as a result of cell lysis. The microscopy shows what appears to be a vesicular localization of catalase under stress; this could be catalase being endocytosed or secreted.

As the Reviewer suggests, identifying Catalase at cellular membranes was unexpected, and we were skeptical as well initially. However, extracellular Catalase has been linked with transformation of cancer cells and characterization of purified Catalase from Drosophila suggested a significant fraction may be membrane bound.

To this end, we have **added more data** highlighting this finding. First, we also show that Catalase re-localizes to membranes in cells adjacent (‘neighbor’) to dying cells in the midgut in vivo using MARCM ‘undead’ clone analysis (**Fig. 5B**). Second, utilizing a neutralizing antibody, we show that Catalase, and thus likely extracellular and/or membrane-associated Catalase, is necessary to prevent cell death spreading and promote cell survival driven by Diedel *in vitro* (**Fig. 6C-D**). Coupled with our other findings (including our unbiased interactome proteomics associated with Diedel-Integrin-ECM interactions at cell membranes), these data further support a role for membrane-associated Catalase, in synergy with Integrin-ECM interactions, in the balance of cell death and survival.

Related to the alternative hypotheses presented by the Reviewer:

We excluded endocytosis from other sources, since endocytosis would likely lead to an increase of intracellular Catalase, and we observe a decrease in intracellular Catalase *in vivo* and *in vitro* when Catalase is at membranes (**Fig. 4 and 5**).

Cell lysis is also very unlikely. The new data provided (‘undead’ clones, **Fig. 5B**) use well characterized genetic tools that are not associated with cell lysis. Second, cells both *in vitro* and *in vivo* that display Catalase re-localization to membranes do not show any molecular features of lytic cells (including loss of membrane structure). Finally, a loss of barrier function induce by significant cell lysis would quickly lead to the death of the flies (**Godenschwege et al Eur J Cell Biol. 2000; Rera et al. PNAS 2012**) In our experimental model utilizing DSS (just as one example), we feed the flies for 2 days with DSS, and let them recover for 2 days (Fig. 6A) without observing any fly death.

2. More proof is required that what we are observing in these figures really is extracellular catalase. All we now have is immunostaining on non-permeabilized tissue.

It is unclear what additional experimental proof is requested. However, we have to respectfully disagree that we only used immunostaining on non-permeabilized tissue to show extracellular catalase. We also used:

1- Endogenously tagged Catalase (Cat-GFP) to visualize Catalase localization after stress in vivo and in vitro (**Fig. 4-5**).

2- We developed a novel assay to measure (*in vivo*) extracellular ROS and specifically extracellular H2O2 (which is the only known catalase substrate). The antibody used only recognizes cysteines modified by H2O2, which promotes dimedone reactivity that can be detected (**Fig. 5C**).

That said, we have **added more data** as described in the response above. First, we show that Catalase re-localizes to membranes in cells adjacent (‘neighbor’) to dying cells in the midgut in vivo using MARCM ‘undead’ clone analysis (**Fig. 5B**). Second, utilizing a neutralizing antibody, we show that Catalase, and thus likely extracellular and/or membrane-associated Catalase, is necessary to prevent cell death spreading and promote cell survival driven by Diedel *in vitro* (**Fig. 6C-D**).

3. The IP for Diedel-interacting proteins in Figure 4B/C and Table S1 is interesting, but there are clearly a very large number of false positives in this analysis. Without validation (for example, conventional co-IP followed by Western blot), this assay doesn't really support much inferential weight. The histogram in 4C also appears to be mislabeled, making its interpretation difficult.

We agree that unbiased interactome proteomics, in this case associated with Diedel-Integrin-ECM interactions at cell membranes, can be difficult to interpret. We have **attempted to clarify this experimentally and in the text.** We show that while Catalase is present at membranes enriched for Diedel-Integrin-ECM interactions, co-immunoprecipitations in native (non-crosslinking) conditions highlight that Diedel and Catalase do not directly interact (**Fig. S6C**), and thus Catalase is likely part of a larger membrane interactome, associated with Integrin-ECM interactions, that shapes cell death and survival responses. We include these data to explain how we became empirically interested by Catalase function at membranes. The synergy between Diedel-Integrin-ECM interactions and membrane-associated Catalase is described in the response to Point 4 below. At this point, we do not believe the Diedel is a molecular driver or anchor that is molecularly/biochemically essential for Catalase re-localization, and that these mechanisms likely work in synergy.

4. It's an important part of the authors' story that Diedel and catalase have a meaningful interaction after tissue damage. However, they don't assay whether the interaction they observe in Figure 4A depends on irradiation or not. This should be shown.

We agree that the initial submission did not clearly integrate Diedel-Integrin-ECM interactions with membrane-associated Catalase. To this end, we have added **new text, discussion, and data (as well as updated the title)** to better highlight the synergy between these functions.

First, we show that adult-specific Diedel over-expression (PplGal4,TubGal80^ts^>UAS-Die) leads to enhanced phospho-ERK immunostaining in midgut enterocytes (**Fig. S5A-D**), suggesting that Diedel-Integrin-ECM interactions likely shape the balance of cell death by, in part, promoting cell survival. Next, we show that while Catalase is present at membranes enriched for Diedel-Integrin-ECM interactions, co-immunoprecipitations in native (non-crosslinking) conditions highlight that Diedel and Catalase do not directly interact (**Fig. S6C**), and thus Catalase is likely part of a larger membrane interactome, associated with Integrin-ECM interactions, that shapes cell death and survival responses. We also show that Catalase re-localizes to membranes in cells adjacent (‘neighbor’) to dying cells in the midgut in vivo using MARCM ‘undead’ clone analysis (**Fig. 5B**). Finally, utilizing a neutralizing antibody, we show that Catalase, and thus likely extracellular and/or membrane-associated Catalase, is necessary to prevent cell death spreading and promote cell survival driven by Diedel *in vitro* (**Fig. 6C-D**). Coupled with our other findings, these data highlight that Diedel-Integrin-ECM interactions likely promote survival of ‘neighboring’ cells, allowing these cells to endure stress an promote Catalase re-localization at membranes to eliminate extra-cellular ROS (**Fig. 5-6**). Thus, these mechanisms likely work in synergy to regulate cell death spreading and ECM degradation in epithelial tissues during stress.

At this point, we do not believe the Diedel is a molecular driver or anchor that is molecularly/biochemically essential for Catalase re-localization (**see Response to Point 3 as well**), and that these mechanisms likely work in synergy. Diedel alone can’t drive Catalase to membranes *in vitro* or *in vivo* (see **Fig. 5C** as an example). Thus, during stress, Diedel-Integrin-ECM interactions likely shift the balance towards cell survival, which helps us visualize Catalase at membranes. Over-expressing or treating cells with Diedel enriches this even further by increasing the number of cells that survive and attenuating cell death spreading. Integrin-ECM interactions and membrane-associated Catalase are likely to work in synergy to influence epithelial resilience.

*That exact/precise molecular mechanism by which Catalase re-localizes to cellular membranes remains unclear, and will be explored in future studies.*

5. Ultimately, the authors want to believe that the relocalization of catalase they observe from the intracellular to the extracellular space is functionally required for control of oxidative damage of ECM; this is explicitly stated in the abstract ("Intracellular Catalase can re-localize to the extracellular membrane and limit ECM damage induced by the amplification of extracellular ROS, a critical adaptive stress response"). However, the requirement for extracellular catalase is not demonstrated.

To more specifically target extracellular Catalase, we **added new data** utilizing a neutralizing antibody (cell-impermeable) to show that Catalase, and thus likely extracellular and/or membrane-associated Catalase, is necessary to prevent cell death spreading and promote cell survival driven by Diedel *in vitro* (**Fig. 6C-D**).

A requirement for Catalase (in general) in regulating epithelial tissue homeostasis (including ECM degradation) was previously provided, utilizing the Catalase inhibitor 3-AT (**Fig. 5 and 6 and Fig. S7A-C**), with the understanding that the inhibitor will not selectively target extracellular Catalase.

Minor point:

The majority of the figures are immunofluorescent images rendered in color-blind unfriendly palettes. Red-green colorblind readers cannot distinguish green from yellow or blue from purple.

We would like to thank the reviewer for bringing this to our attention. Moving forward, we will take this into strong consideration as we acquire immunofluorescent images, especially using blue and purple in the same frame.

Rev.3:

In this manuscript, the authors characterize a secreted protein, Diedel (die), which they have identified in previous work, and link its activity to resilience of epithelial cells, mostly at the adult midgut. Diedel is secreted from fat body and inhibits apoptosis-induced regulated cell death (RCD). Here, they generate two mutants of die and find that the integrity of the epithelial architecture and integrity of the midgut is disrupted. By using a tagged transgene they localize Diedel to the basement membrane of the midgut. The authors then provide evidence that Diedel interacts with the integrin Mys via its RGD motif and is required for attaching epithelial cells to the extracellular matrix. The authors then move on to examine how Diedel links the integrity of the epithelium to its control of apoptosis-induced RCD. In a trans-well cell culture system they show that Diedel inhibits apoptosis-induced RCD through maintaining the ECM-integrin interaction. Finally, the authors identify Catalase as an interacting protein of Diedel in UV-treated S2 cells. What follows is - in my mind - a rather confusing attempt to link redox balance mediated by Catalase with tissue resilience after stress.

Overall, this manuscript has strengths and weaknesses. A major strength is the finding that Diedel can act as a ligand for the integrin Mys. However, other sections of the manuscript are rather weak, vague and often confusing.

1. In figure 1, the authors claim that Diedel localizes to the basement membrane. However, they never use any marker of the basement membrane to directly demonstrate this localization. They also claim that in die mutants, enterocytes detach from the basement membrane. Again, without proper markers, these conclusions are hard to justify.

We have **added new data** showing that Deidel co-localizes with Laminin B1 at the basement mebrane. We assessed Diedel localization (utilizing Die^P^-DieV5 flies) within a genetic background that endogenously expresses a multi-tag-GFP Laminin B1. The basal lamina is the layer of the ECM that is in direct contact with epithelial cells, it is formed by a self-assembling network of Laminin heterotrimers consisting of an alpha, beta and gamma subunit. Immunostaining confirmed colocalization of DieV5 with LanB1 at the midgut epithelium basement membrane (**Fig. S1G**).

To address the concerns about properly marking enterocytes to assay cell detachment, we have **added new data** showing detached cells (in Diedel mutants) immunostain positive for Pdm1, a commonly used enterocyte cell marker **(Fig. S1E** and **Korzelius et al. EMBO J. 2014)**

2. The description of the cleaved Dcp-1 staining in figure 3 was not well presented. First, the authors seem to believe that caspases are nuclear (see lines 319 and 328). Caspases can be nuclear, but they are largely cytosolic. So, the cDcp1 staining should by mostly cytosolic. The membrane localization of Dcp-1 is interesting and there is precedence for that, but to consider the possibility that a cytosolic protein can interact with the extracellular basement membrane (line 321) is rather strange

We **have attempted to clarify** this concern in the text. In line 321, we actually do not mention the ‘extracellular basement membrane’ and instead referred to the ‘cellular membrane’. Based on the co-localization of Dcp-1 and Dlg (which we now understand the Reviewer didn’t have access to), we meant to imply that ‘cellular membrane’ would more specifically be the intracellular membrane. However, because so much of the manuscript focuses on the ECM and basement membrane, we can understand that this text needs additional description. We have now added ‘intracellular membrane’ to the text to provide clarity.

Second, although the authors show increased caspase activity in die mutants, they do not show whether there is more apoptosis . How does TUNEL labeling or other apoptosis assays look like in die mutants?

We have **added new data** showing that indeed, TUNEL staining is elevated in Diedel mutant animals (**Fig. S3I**)

Furthermore, in our previously published manuscript, we extensively used the TUNEL assay to characterize cell death/apoptosis in the midgut epithelium in response to Diedel attenuation or over-expression (**Mlih et. al., PLoS Biology, 2018**).

3. The co-localization data of Dcp1 with Dlg1 in figure S3E was not present in my copy of the manuscript.

We hope this is rectified in the revised version of the manuscript. To clarify, the Dcp-1 and Dlg co-localization immunostains are now provided in **Fig. S4B**.

4. The section about the characterization of Catalase is very confusing. The authors state that they identified cytosolic Catalase as an interacting protein of extracellular Diedel. I understand that similar to Dcp1 above, a cytosolic protein can be membrane localized under certain circumstances. However, how can extracellular Diedel interact with intracellular protein. In lines 391/2, they state that Catalase is extra-cellular and the non-permeabilized conditions of the experiments further supports the extracellular location. At another point in the manuscript (lines 415/6), the authors state that Catalase acts on extracellular ROS. How can that be? Can Catalase cross membranes? I also find the conclusion of this section that "these molecular changes underly resilience mechanisms in epithelial tissues" (lines 422/3) very vague and over-interpreted.

We agree that the initial submission did not clearly integrate Diedel-Integrin-ECM interactions with membrane-associated Catalase. To this end, we have added **new text, discussion, and data (as well as updated the title)** to better highlight the synergy between these functions.

First, as the Reviewer suggests, identifying Catalase at cellular membranes was unexpected, and we were skeptical as well initially. However, extracellular Catalase has been linked with transformation of cancer cells and characterization of purified Catalase from Drosophila suggested a significant fraction may be membrane bound.

For additional precedent, resident ER proteins can re-localize to extracellular space during stress to rebuild basement membranes (**Ilani et al. Science 2013**), and in Drosophila, CabP1 protein (another ER resident and a protein we identified in our unbiased proteomics as associated with Diedel-Integrin interactions at extracellular membranes, **Fig. 4C**) can re-localize to extracellular space in response to cell death (). Specifically related to Catalase, this protein can readily cross peroxisome membranes into the cellular cytosol (**Okada et al J. Biol. Chem. 2012**) Thus, shuttling specific proteins across various types of membranes is a common feature of stress responses.

Related to **new data provided in this revision**, we show that adult-specific Diedel over-expression (PplGal4,TubGal80^ts^>UAS-Die) leads to enhanced phospho-ERK immunostaining in midgut enterocytes (**Fig. S5A-D**), suggesting that Diedel-Integrin-ECM interactions likely shape the balance of cell death by, in part, promoting cell survival. Next, we show that while Catalase is present at membranes enriched for Diedel-Integrin-ECM interactions, co-immunoprecipitations in native (non-crosslinking) conditions highlight that Diedel and Catalase do not directly interact (**Fig. S6C**), and thus Catalase is likely part of a larger membrane interactome, associated with Integrin-ECM interactions, that shapes cell death and survival responses. We also show that Catalase re-localizes to membranes in cells adjacent (‘neighbor’) to dying cells in the midgut in vivo using MARCM ‘undead’ clone analysis (**Fig. 5B**). Finally, utilizing a neutralizing antibody, we show that Catalase, and thus likely extracellular and/or membrane-associated Catalase, is necessary to prevent cell death spreading and promote cell survival driven by Diedel *in vitro* (**Fig. 6C-D**). Coupled with our other findings, these data highlight that Diedel-Integrin-ECM interactions likely promote survival of ‘neighboring’ cells, allowing these cells to endure stress an promote Catalase re-localization at membranes to eliminate extra-cellular ROS (**Fig. 5-6**). Thus, these mechanisms likely work in synergy to regulate cell death spreading and ECM degradation in epithelial tissues during stress.

At this point, we do not believe the Diedel is a molecular driver or anchor that is molecularly/biochemically essential for Catalase re-localization, and that these mechanisms likely work in synergy. Diedel alone can’t drive Catalase to membranes *in vitro* or *in vivo* (see **Fig. 5C** as an example). Thus, during stress, Diedel-Integrin-ECM interactions likely shift the balance towards cell survival, which helps us visualize Catalase at membranes. Over-expressing or treating cells with Diedel enriches this even further by increasing the number of cells that survive and attenuating cell death spreading. Integrin-ECM interactions and membrane-associated Catalase are likely to work in synergy to influence epithelial resilience.

*That exact/precise molecular mechanism by which Catalase re-localizes to cellular membranes remains unclear, and will be explored in future studies.*

5. The trans-well cell culture experiments are quite interesting. How is apoptosis transmitted from UV-treated cells to untreated cells? Is there a death signal? The authors must be aware of the work by Perez-Garijo et al. (2013) in eLife which describes apoptosis-induced apoptosis in imaginal discs. In that work, Eiger was identified as a death ligand. Does that apply here, too?

TNF/Eiger was one of the first factors we explored to understand the role of Diedel in antagonizing cell death (based on Perez-Garijo et al., eLife, 2013). However, we found no role for Eiger (in vivo or in vitro) in shaping Diedel-dependent cell death regulation utilizing a multitude of assays. We **have included** one assay in updated version of Fig. S3, see **Fig. S3F**). This also is likely to underly significant differences in cell death regulation between the developing animal (developing epithelial tissues such as imaginal discs) and post-eclosion adult animals (such as the midgut epithelium), which is apparent in both TNF/Eiger signaling and Wnt signaling (**Eiger: Doupé et al. PNAS 2018 vs Igaki et al EMBO J. 2002; Wnt: Cordero et al. Mech Dev. 2004 vs Lin et al. 2008 Nature**).

To help clarify this point, we have also **added data to show** that Catalase (and likely membrane-associated Catalase due to the use of a non-permeable neutralizing antibody) is required for Diedel-dependent effects on cell death and cell death spreading (**Fig. 6C-D**). Since the only known function of Catalase is to eliminate hydrogen peroxide, it is likely that the secondary signal coming from the dying cell are ROS or ROS-generating factors. Furthermore, it was shown in another study that dying Drosophila cells recruit the oxidase DUOX at cellular membranes to generate extracellular ROS, and that by expressing a human secreted Catalase, cell death inducing proliferation can be attenuated (**Fogarty** **et al. Current Biology 2016**).

We have also attempted to clarify all of this in the text.
