# Supplementary material for: Integrin–ECM interactions and membrane-associated Catalase cooperate to promote resilience of the Drosophila intestinal epithelium
Source: PLoS Biol. 2022 May 6;20(5):e3001635. doi: 10.1371/journal.pbio.3001635 (PMC9116668; doi:10.1371/journal.pbio.3001635)
Supplement: Attachment — Submitted filename: Response to Reviewers.docx [file pbio.3001635.s013.docx]

Reviewers' comments

Rev. 1:

The authors have attempted to solve many concerns I raised during the initial round of review. Thanks to the new data added to the manuscript, now it seems more convincing to conclude what authors tried to show.

Rev. 2:

This is a re-review of the manuscript now titled "Synergy between Integrin-ECM interactions and membrane-associated Catalase promotes resilience of the Drosophila intestinal epithelium" by Mlih and Karpac.

The prior version of this manuscript was odd: the first part was a solid exploration of integrins as receptors for Diedel and the second part was a description of the claim that catalase is secreted under stress and interacts with Diedel to protect the epithelium. The connection between the two parts was tenuous. The connection between the two parts has now become more tenuous because the authors no longer think that Diedel and catalase physically interact. It is even less clear how we are to interpret the proteomic experiment in 4A given that the authors no longer believe that there is a direct physical interaction between Diedel and catalase. The figure legend here still reads "Mass spectrometry of gel-based pulldown analysis (Diedel-interacting proteins)".

In both versions of the manuscript, Diedel-Catalase interactions are never described as direct, and they were never described as a direct physical interaction (interactions can be physical direct or indirect). In the revised version, we did additionally clarify that this interaction is very likely to be indirect based on the nature of Diedel function and the biochemistry used in the pull-down analysis. We do agree, however, that labeling these experiments in the Figure Legend as “Mass spectrometry of gel-based pulldown analysis (Diedel-interacting proteins)” was confusing, and we regret not removing it earlier. It has now been removed and redescribed in the Figure Legend.

In this further revised version, we have additional clarity to the text (Abstract and Results) to better describe these findings conceptually and experimentally (including details in the biochemistry of the pull-downs) - **highlighted in blue**.

One of the most surprising parts of the first manuscript was the claim that catalase is present and required in the extracellular space to protect tissue from stress. This would be very important if shown. However, the experiments in the prior version of the paper were not strong in this regard and the authors do not give a significantly stronger reason to believe that this is the case. The added functional experiment (blocking extracellular catalase with an antibody) requires controls. The antibody used would not be predicted to interact strongly with Drosophila catalase, based on the location of the epitope, and it's unclear whether it would block catalase function even if it were bound. This experiment would require that these points be clarified. It would also require control treatment with an isotype control antibody to eliminate other kinds of artifacts.

Related to the neutralizing antibody experiments, we provided genetic analysis to show that the antibody targets Drosophila Catalase and *in vitro* analysis to show it directly impacts Catalase function, and these experiments have been done previously as cited in the text (although in mammalian cells). We do agree that an isoptype control would have enhanced the interpretation. In light of this, we have removed the neutralizing antibody experiment from the manuscript.

The oxidized cysteine experiments are interesting but also do not directly require extracellular catalase—this effect could be indirect.

To the best of our knowledge, Catalase is the only enzyme in Drosophila that can decompose H2O2. We used this technique because the oxidation of cysteine is H2O2 dependent, which thus allows an ***indirect*** measurement of extracellular H2O2 (especially when coupled with Catalase attenuation) in our various biochemical/genetic models. This is exactly how it is described in the text, and is just one of many experiments (direct or indirect) that we used to support our hypothesis.

The most obvious way for catalase to end up in the extracellular space would be fusion of peroxisomes with the plasma membrane. This would be very interesting but is not explored. The references given in support of this possibility in the response to reviewers are not salient to this question, or to the larger question of how catalase ends up outside the cell; they all deal with proteins in the ER or Golgi (known secretory compartments) being secreted in a condition or cell-type dependent way.

This was not mentioned as a concern in the original review, but as we mentioned in the discussion of both versions of the manuscript, we agree that the mechanism by which Catalase re-localizes to membranes is of interest. However, we currently do not have a distinct testable hypothesis for this re-localization, and this will likely require a degree of *in vivo* genetic screening in Drosophila. The hypothesis of ‘peroxisome re-localization to the membrane’ was our initial hypothesis as well, but we tested this using a Peroxisome membrane marker and did not observe any changes in peroxisome localization.

The reference in the text is not meant to explain how catalase goes to the membrane in our experimental model, but is given to highlight that Catalase re-localization was describe previously in another study using cancer cells (to give some precedent for the surprising finding).

The reference to re-localization of ‘stress proteins’ to membranes during stress (mainly from the ER Golgi) was provided not in response to a question of mechanism, but in response to the reviewers concern that cytosolic proteins (like Catalase) can end up at all in membranes after stress. This is also referenced as an example of cytosolic proteins/enzymes being re-located to the basement membrane to help repair/rebuild the ECM during stress.

There may be something in the second half of this paper that is very important. It deserves to be analyzed carefully and thoroughly, not appended to a perfectly fine paper on Diedel receptors. Demonstration that integrins are Diedel receptors would appear to be a perfectly sufficient research update in its own right.

We respectfully disagree with the ‘appended’ comment. The data related to Catalase re-localization was acquired and interpreted with the same rigor as applied to the Diedel-Integrin interaction data. This includes developing novel tools to assess Catalase activity, as well as integrating various (and sometimes complex) genetic and biochemical models.

Rev. 3:

I am pleased with the revision of this manuscript. The authors have addressed all comments and weaknesses, the writing has improved and they have added new data to further support their statements. It is an interesting paper with important conclusions.

However, I have a few corrections about the statements the authors made about reference 11: In the discussion (lines567-570), the authors state that ref. 11 used genetically induced 'undead' cells. That is not true. Ref. 11 postulates that dying enterocytes in the adult midgut undergo a transiently undead state in the course of dying. This state is not genetically induced (like by coexpression of p35). Furthermore, in the same context, the authors state that ref. 11 showed that Duox (via Myo1D) localizes to the basement membrane. Again, that was not shown in ref. 11. Duox is a transmembrane protein sitting in the plasma membrane. What was shown in ref 11 is that the caspase Dronc is localized to the plasma membrane via Myo1D. These incorrect statements need to by corrected by the authors.

We apologize for these errors and have corrected this in the discussion of text (**highlighted in blue**).
